# Transparent Ferroelectric Capacitors on Glass

**DOI:** 10.3390/mi8100313

**Published:** 2017-10-20

**Authors:** Daniele Sette, Stéphanie Girod, Renaud Leturcq, Sebastjan Glinsek, Emmanuel Defay

**Affiliations:** Luxembourg Institute of Science and Technology, Department of Materials Research and Technology, 41 rue du Brill, Belvaux L-4422, Luxembourg; daniele.sette@gmail.com (D.S.); stephanie.girod@list.lu (S.G.); renaud.leturcq@list.lu (R.L.); sebastjan.glinsek@list.lu (S.G.)

**Keywords:** transparent piezoelectrics, glass substrate, chemical solution deposition

## Abstract

We deposited transparent ferroelectric lead zirconate titanate thin films on fused silica and contacted them via Al-doped zinc oxide (AZO) transparent electrodes with an interdigitated electrode (IDE) design. These layers, together with a TiO_2_ buffer layer on the fused silica substrate, are highly transparent (>60% in the visible optical range). Fully crystallized Pb(Zr_0.52_Ti_0.48_)O_3_ (PZT) films are dielectrically functional and exhibit a typical ferroelectric polarization loop with a remanent polarization of 15 μC/cm^2^. The permittivity value of 650, obtained with IDE AZO electrodes is equivalent to the one measured with Pt electrodes patterned with the same design, which proves the high quality of the developed transparent structures.

## 1. Introduction

In parallel to the development of technologies on silicon, there is a large panel of new functions dedicated to transparent substrates and more specifically to glass [1]. This should involve completely transparent functional layers such as transistors [2] and sensing elements [3]. Transparent pixels or photovoltaic devices have already been published in the literature [4]. Similarly to the More than Moore trend, one can think of developing new devices on glass with optical or mechanical coupling in order to multiply applications. For instance, it has recently been suggested that haptic functions can be added on mobile phone screens through piezoelectric ceramics [5]. It would be ideal to achieve completely transparent stacks on glass so as not to perturb light transmission when it comes to adding new functionalities to screens. We will eventually target transparent piezoelectric stacks in order to fabricate haptic devices. More specifically, we want to study lead zirconate titanate (PZT) films, piezoelectric films with one of the highest reported piezoelectric coefficients, deposited on glass. Moreover, we aim at suppressing the bottom electrode in order to potentially improve the overall transparency. It induces that top electrodes will be interdigitated. Our top choice of electrode is Al-doped zinc oxide (AZO) because of its optical transparency and electrical conductivity [6]. In addition, it does not contain any rare and/or harmful components such as indium in indium tin oxide (ITO) electrodes.

The deposition of PZT on glass substrates, unlike PZT films on silicon, has barely been studied. All examples with electrical results involved a bottom electrode, which was made of Pt or transparent conductive oxides, such as ITO [7] or fluorine-doped tin oxide (FTO) [8]. Khodorov et al. deposited La-doped PZT via the sol–gel method on tin oxide-coated glass substrate [9]. The reflection in the visible range for 130-nm-thick La-doped PZT (PLZT) did not exceed 20%, and no electrical results were performed. Ohno et al. showed that 1.1-µm-thick PZT with compositions close to the morphotropic phase boundary (MPB, corresponding to Zr/Ti = 52/48), deposited via chemical solution deposition (CSD) on soda lime glass and covered with CSD ITO exhibited a dielectric permittivity *ε_r_* close to 1000, a remanent polarization as large as 36 µC/cm², and a longitudinal piezoelectric coefficient *d*_33_ reaching 120 pC/N after thermal treatment at 600 °C [10]. The transmittance of the stack without top electrode was around 60% in the visible optical range, which was a substantial improvement with respect to Khodorov et al. Liu et al. [11] obtained very similar results on Co-doped PZT also deposited via CSD on an ITO/glass substrate. More recently, Bayraktar et al. [12] focused on the crystalline quality of PZT films grown on glass and reported epitaxial growth of MPB PZT by utilizing Ca_2_Nb_3_O_10_ and Ti_0.87_O_2_ nanosheets as crystalline buffer layers, combined with ITO or Pt/ITO as bottom electrode. Another interesting approach leading to epitaxial films has been proposed by Terada et al. [13], who first grew PZT on MgO and then transferred the layers to glass substrates, though Pt electrodes have been utilized. The unique example of a fully transparent and electrically functional PZT-based stack on glass substrates was published in 2007 by Uprety et al. [14]. The studied stack was ITO/LNO/PZT/LNO/ITO (where LNO stands for LaNiO_3_) on glass. The PZT was 90-nm-thick, and the overall transparency of the final device capacitor was around 50% before the deposition of the top electrode. In this paper, we propose to improve the overall transparency of the same kind of MPB PZT stack by suppressing the bottom electrode and adopting an in-plane interdigitated electrode (IDE) configuration, similar to what has been performed on silicon more than a decade ago [15].

## 2. Experimental Section

A 2”-diameter and 500-µm-thick substrate made of optical grade fused silica with roughness Ra <1 nm has been used. A buffer layer was needed between glass and PZT to avoid cracks. Here, this buffer layer was made of 20-nm-thick oxidized titanium deposited via evaporation at 25 °C and annealed at 700 °C in air. Such high temperature infers the complete oxidation of Ti, which stabilizes titanium oxide with respect to the subsequent steps. PZT with MPB composition—meaning Zr/Ti = 52/48—was deposited by spin coating three successive layers of “PZT-E1” commercial sol precursors from MMC (Mitsubishi Materials Corporation, Tokyo, Japan). Each of the three PZT layers was spun, dried at 130 °C for 5 min, and pyrolyzed at 350 °C for 5 min on hot plate in air. A final annealing step performed in a box furnace at 700 °C in air for 30 min induced the crystallization of the 200-nm-thick PZT film in the desired perovskite structure. The 115-nm-thick AZO IDE electrodes were then deposited via atomic layer deposition and patterned through a lift-off process using lift-off resist (LOR) Shipley resist in order to generate an undercut below a resist layer (1813 Shipley). Reference samples made of sputtered 100-nm-thick Pt electrodes with the same IDE design were also realized by standard lithography/ion beam etching process. The absolute values of electric field *E*, relative dielectric permittivity *ε*’ and polarization *P* were extracted from the measurements as recently described by Nigon et al. [16]. Because the gap and width of the fingers are significantly larger than the thickness of the film (*a*, *b* >> *t_f_*), the effective gap *a* + Δ*a* (Δ*a* = 1.324 × *t_f_*) was used in the calculations.

X-ray diffraction patterns were obtained with a Panalytical diffractometer in *θ*-2*θ* configuration, - 2*θ* being the measurement angle of the detector with respect to X-ray incident beam on the sample at angle *θ*. The light transmittance of the stack was measured on a TECAN (Tecan Group Ltd., Zürich, Switzerland) absorbance instrument. The Agilent atomic force microscope (AFM) was used to measure root-mean-square (RMS) surface roughness. The permittivity-electric field (*ε’*-*E*) curves and polarization-electric field (*P*-*E*) loops were collected with an Aixacct set-up.

## 3. Results

**Figure 1:**
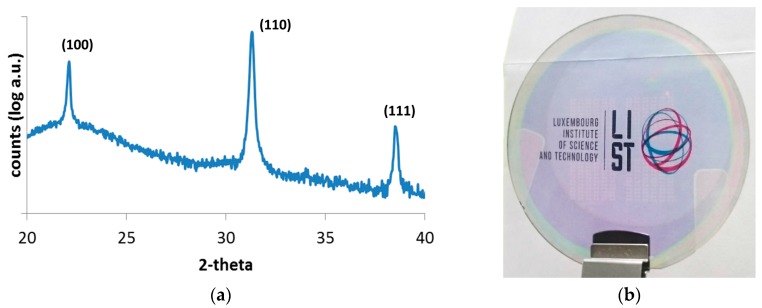
(**a**) The *θ*-2*θ* pattern of 200-nm-thick Pb(Zr_0.52_Ti_0.48_)O_3_ (PZT) film deposited by spin coating on TiO_2_/fused silica and crystallized at 700 °C. All peaks correspond to the perovskite structure; (**b**) Optical image of a 2’’-diameter wafer coated with 20 nm of TiO_2_, 200 nm of PZT, and 115 nm of patterned Al-doped zinc oxide (AZO), the latter being visible in the central area of the wafer. TiO_2_ exhibits a purplish color. Note that the logo is visible through the glass wafer.

Figure 1a shows the X-ray diffraction pattern of crystallized PZT films on glass. The perovskite structure is the only visible phase. No secondary phase such as pyrochlore has been detected. There is no preferred crystalline orientation as one could expect on an amorphous substrate such as fused silica. The RMS value obtained from 5 × 5 μm^2^ areas by AFM is 2.0 nm. Figure 1b shows a top view of the transparent finalized substrate—fused silica/TiO_2_/PZT/AZO—as observed with optical microscopy. Note that the logo is not printed on the wafer but is visible through the latter. PZT film is homogeneous and did not encounter cracks during the fabrication process. Our experiments have shown that a buffer layer of TiO_2_ with a minimum thickness of 10 nm is mandatory to avoid cracks. For thinner layers or no buffer layer at all, PZT crystallizes but exhibits cracks. 

**Figure 2:**
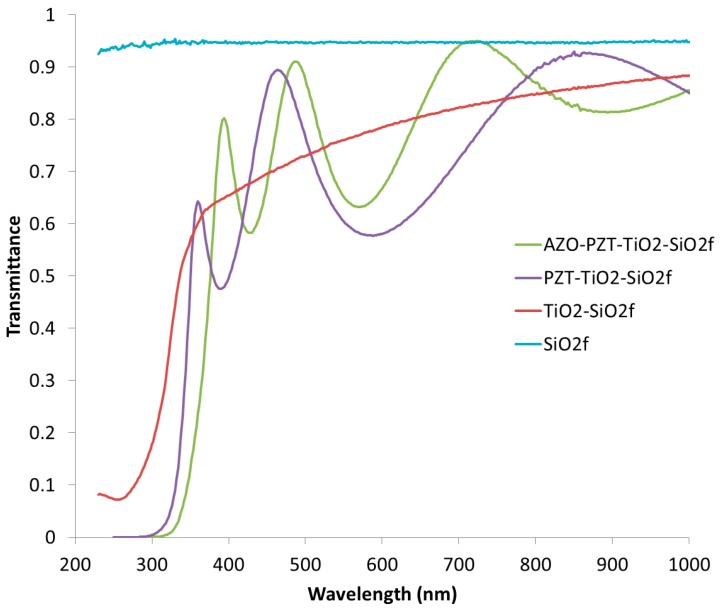
Optical transmission spectra from 232 nm to 1000 nm measured on the successive stacks of AZO/PZT/TiO_2_/fused silica.

The main drawback of TiO_2_ is that it induces a purplish color that limits the device’s transparency, as can be seen in Figure 1b. Fused silica exhibits transmittance beyond 93% in the visible spectral range (cf. Figure 2). This transmittance decreases down to 65% around *λ* = 400 nm after TiO_2_ has been deposited. Adding PZT creates Fabry–Perot oscillations but does not strongly impact the overall transmittance. It also shifts the minimum wavelength that can cross the wafer from 300 nm with TiO_2_ to 340 nm if one takes 20% transmittance as a reference. The final 115-nm-thick AZO slightly improves the stack transmittance probably because of the index matching effect. Indeed, the ZnO refractive index is around 2.0, whereas that of PZT is larger, around 2.4. Consequently, capping PZT with ZnO-based material smoothens the gap and therefore acts as an index matching layer. AZO main drawback is that it stops transmittance below 360 nm. All in all, the stack transmittance is higher than 60% in the range from 400 nm to 1000 nm. As mentioned in the introduction, Uprety et al. reported 50% transmittance on their transparent piezoelectric stack involving ITO as the bottom electrode [14]. Therefore, our strategy to suppress the bottom electrode helps in improving transparency.

**Figure 3:**
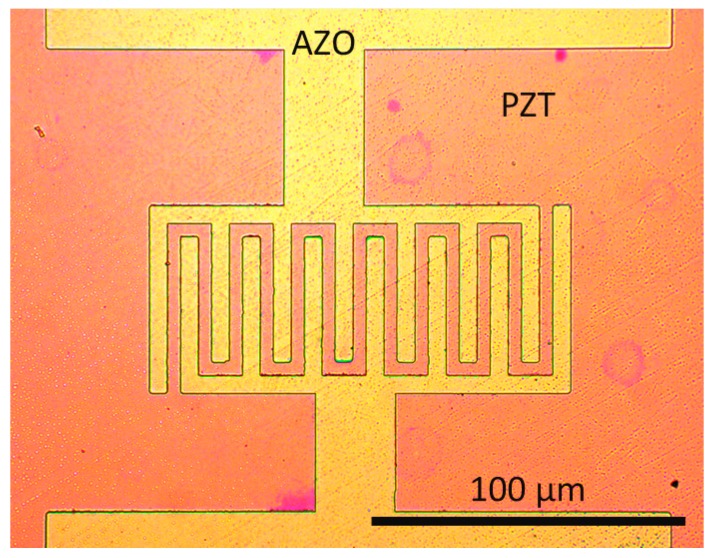
Top-view optical micrograph of a final device showing patterned AZO IDE top electrodes. Here, the gap between subsequent fingers is 5 µm, and each finger is 5-µm-wide.

Making electrically functional transparent piezoelectric devices without bottom electrode infers that both electrodes have to be processed on the top of the piezoelectric layer. To do so, we patterned AZO IDE electrodes as shown in Figure 3. Various gaps (5, 10, and 20 µm) were processed. Although AZO is not as conductive as ITO (typical sheet resistance of 250 Ω^2^ for 100-nm-thick films in our case), it only involves abundant and low-cost metals (aluminium and zinc).

**Figure 4:**
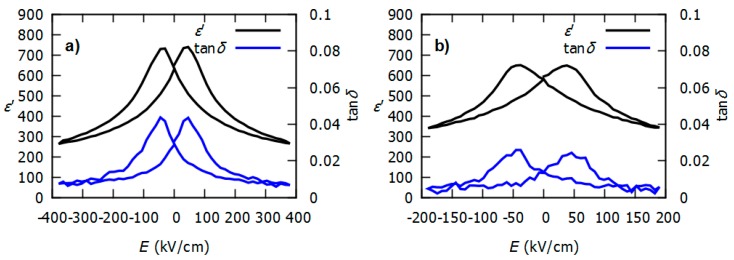
Relative permittivity *ε’* and tan*δ* at 1 kHz versus DC electric field *E* of a 7-tooth IDE structure with a 5 µm gap. Electrodes are made of (**a**) AZO and (**b**) Pt. For permittivity calculation, the stray capacitance was subtracted from the measured capacitance.

Figure 4a shows the relative permittivity *ε’* and dielectric losses tan*δ* versus the DC electric field of the 7-tooth IDE structure reported in Figure 3. Both curves display the typical hysteretic behavior of ferroelectric films, with respective zero-field values of permittivity and tan*δ* of 650 and 0.03. Contrary to what is generally observed in metal–insulator–metal structures (MIM), the curves are very symmetrical with respect to the Y-axis. This is a direct consequence of the symmetrical IDE structure that has been processed with the same metal. The losses are very low—below 1%—at voltages as large as 400 kV/cm. It evidences the good quality of PZT, but also the compatibility of AZO electrodes with PZT in this IDE structure.

Figure 4b represents the same characterization on the same IDE structure, though with Pt electrodes. The difference lies in the maximum electric field applied, which is only 200 kV/cm with Pt. Indeed, this structure was unable to withstand larger field because of the ion milling etching step that weakened PZT and/or induced Pt re-deposition. It ended up with strong extrinsic leakage at a high electric field. In Figure 4, one can spot that permittivity values are very similar for both AZO and Pt electrodes. The latter is considered as the reference metal for PZT. Therefore, it shows that AZO stands for a convincing transparent alternative to Pt in order to contact PZT structures.

**Figure 5:**
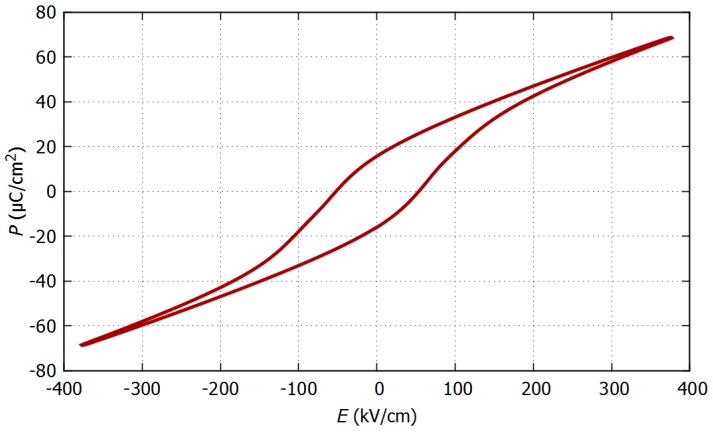
Polarization-electric field (*P*-*E*) loop of the 200-nm PZT on glass with AZO 5-µm-gap IDE electrodes. The measurement was performed at 100 Hz.

Finally, a clear ferroelectric behaviour is observed on the polarization-electric field *(P-E)* loop of PZT on glass with AZO electrodes, as shown in Figure 5. The extracted value of remanent polarization *P_r_* is 15 μC/cm^2^, which is approximately half the value reported for 500-nm-thick (100) MPB PZT on Si with Pt IDE electrodes, but comparable to its counterpart in MIM geometry [17]. The calculated coercive field *E_c_* is 52 kV/cm. The slanted shape of the loop is a measurement artefact due to relatively large stray capacitance compared to the total capacitance value (measured on IDE structures and shown in Figure 3). We wanted to ensure that this tilted loop was not induced by AZO’s rather high resistivity, so we compared it with Pt top electrodes patterned with the same design. Both loop shape and coercive voltage are very similar to what has been observed with AZO electrodes. In addition, we found that AZO equivalent resistance of Figure 3 is around 700 Ω. This impedance is negligible compared to PZT, which lies in the GΩ range (*C*~1 pF) during the *P-E* loop collection performed at 100 Hz. Consequently, AZO’s higher resistance has no significant influence on the *P-E* loop’s shape.

## 4. Conclusions

In this paper, we have shown that it is possible to make functional transparent ferroelectric structures with PZT and IDE AZO electrodes. The measured permittivity, dielectric losses, and polarization values of 650, 0.03, and 15 μC/cm^2^, respectively, have confirmed typical ferroelectric behavior, which is a pre-requisite of piezoelectric ferroelectric materials such as PZT. The main advantage of the proposed structure is the absence of bottom electrode, which implies improved transparency of the whole PZT stack (>60% in the visible range) if one compares with Uprety et al.’s [14] study with the ITO bottom electrode.
